# The Expression of Forkhead Box P3 T Regulatory Lymphocytes as a Prognostic Factor in Malignant Melanomas

**DOI:** 10.3390/ijms25126377

**Published:** 2024-06-09

**Authors:** Vlad Alexandru Gâta, Andrei Pașca, Andrei Roman, Maximilian Vlad Muntean, Dragoș Ștefan Morariu, Eduard Alexandru Bonci, Constantin Dina, Loredana Ungureanu

**Affiliations:** 1 Department of Surgical Oncology and Gynecologic Oncology, “Iuliu Hațieganu” University of Medicine and Pharmacy, 400012 Cluj-Napoca, Romania; 2 “Prof. Dr. Ion Chiricuță” Institute of Oncology, 400015 Cluj-Napoca, Romania; 3 Department of Radiology, “Iuliu Hațieganu” University of Medicine and Pharmacy, 400012 Cluj-Napoca, Romania; 4 Department of Plastic and Reconstructive Surgery, “Iuliu Hațieganu” University of Medicine and Pharmacy, 400012 Cluj-Napoca, Romania; 5 “Champalimaud“ Research and Clinical Centre, 1400-038 Lisbon, Portugal; 6 Department of Anatomy, Faculty of Medicine, Ovidius University, 900470 Constanta, Romania; 7 Department of Dermatology, “Iuliu Hațieganu” University of Medicine and Pharmacy, 400012 Cluj-Napoca, Romania; 8 Department of Dermatology, Emergency County Hospital Cluj-Napoca, 400006 Cluj-Napoca, Romania

**Keywords:** FOXP3, malignant melanoma, metastasis, prognosis, tumor-infiltrating lymphocytes

## Abstract

Since transcription factor Forkhead Box P3 (FoxP3) was identified as a specific regulatory T cell (Treg) marker, researchers have scrutinized its value as a potential novel therapeutic target or a prognostic factor in various types of cancer with inconsistent results. The present analysis was performed to assess the influence of Treg FoxP3 expression on the prognosis of primary melanoma and to evaluate the correlations with various clinicopathological prognostic factors. We analyzed all eligible patients with stage pT3 primary malignant melanomas treated in a tertiary cancer center. Immunohistochemical staining for Treg FoxP3 expression was performed on retrospectively identified paraffin blocks and subsequently correlated with the outcomes of the patients. A total of 81% of the patients presented a positive Treg FoxP3 expression, being correlated with a higher risk of lymph node metastasis, tumor relapse, and death. Moreover, positive expression was statistically associated with a shorter OS. The tumor relapse rate was estimated at 36.7%. A positive expression of Treg FoxP3 and lymph node metastasis were associated with a higher risk of death based on multivariate analysis. Treg FoxP3 expression may be used as an independent prognostic factor in patients with malignant melanoma to evaluate tumor progression and survival.

## 1. Introduction

Malignant melanoma, the cancer of melanocytes, represents the principal cause of mortality among skin neoplasias, accounting for up to 60% of skin cancer-related deaths, with boosting incidence and mortality rates worldwide [1]. Moreover, in the past decade, the incidence has risen among people aged 20–35, currently being rated as the most frequent cancer affecting young people [2,3]. Despite the countless public health campaigns and the efforts undertaken in the last decade regarding its diagnosis and treatment, the long-term prognosis has not been significantly enhanced [4]. Hence, the effective management of this disease should include prevention and the early diagnosis of high-risk patients in order to decrease mortality. Nevertheless, due to fierce investigations and numerous clinical trials undertaken in recent years, modern therapies eloped and revealed excellent results, statistically reducing mortality and improving the overall survival of the patients. These therapies include mitogen-activated protein kinase and BRAF inhibitors for a subset of well-selected patients, as well as immunotherapy (anti-PD1, PD-L1, and anti-CTLA4 agents) for the treatment of metastatic or unresectable melanoma [5,6,7,8,9,10,11,12]. Regardless, a small cohort of patients, through different immunological mechanisms, may develop a resistance to immunotherapy, either primarily or after a promising initial response, leading to tumor progression or tumor relapse [13,14,15].

Malignant melanoma is a very aggressive tumor with a high potential for metastasis, both through hematogenous and lymphatic pathways. Regarding visceral metastasis, even though it is the third most common source of brain metastasis, it has the highest tendency to metastasize [16], the mortality being related to the metastatic visceral spread to different organs. Moreover, studying the pathogenesis of this disease, researchers heeded that tumor progression is favored by a complex interaction between tumor cells and the host’s immune response [17], deeming malignant melanoma an immunogenic tumor that quickly stimulates immune reactions in the host’s organism [18,19,20]. In addition, melanoma was pinpointed as having the highest mutational load of all malignant tumors [21,22]. These mutations occur predominantly within the mitogen-activated protein kinase pathway (MAPK) or along the PI3K/AKT pathway through the activation of the following three oncogenes that melanoma hosts: Braf, N-Ras, and c-Kit [23,24]. In this regard, the immune response has recently gained awareness to identify immune evasion mechanisms and contain tumor growth.

The immune system maintains homeostasis by protecting the host from infections with different pathogens and exerting an antitumoral immune response. Consequently, the value of tumor-infiltrating lymphocytes has been intensively examined in different types of cancer; in melanoma, these cells may prevent proliferation and tumor progression and represent an independent prognostic factor for lymph node metastasis and overall survival [25,26,27,28,29]. Moreover, TILs comprise a heterogeneous group of cells that cultivate an immune response as T effector cells and functional and regulatory T cells, playing different roles in the tumor microenvironment [30]. As the plurality of TILs expresses a CD3+ phenotype, these can be divided into cytotoxic T cells (CD8+), memory T cells (CD45RO+), and regulatory T cells (CD4+CD25+) [31]. CD8+ T lymphocytes are considered the foremost antitumor effector cells, with studies implying that their presence is associated with a more reasonable prognosis in different types of cancer [32,33]. Regulatory T cells (Tregs) are a small subpopulation of CD4+ T cells (accounting for 5–10% of T cells in the periphery) involved in upholding immunological tolerance for self and non-self and, therefore, stemming autoimmune diseases [34].

Nevertheless, Tregs also exert an immunosuppressive effect by inhibiting antitumor immunity through the suppression of the antitumor cytotoxic T cells, precisely by inhibiting the activation and differentiation of CD4+ T helper cells and CD8+ cytotoxic T cells [35,36]. Since 1970, when the concept of suppressor T cells was identified for the first time, myriad studies have been conducted to characterize CD4+ T cells and better understand the immune system’s role in developing autoimmunity. Only 20 years later, in 1990, an in vivo study proved the existence of suppressor T cells against tumor immunity. Since then, this has become a topic of noteworthy interest [37,38,39,40]. Subsequently, in 2003, the transcription factor Forkhead Box P3 (FoxP3) was identified as the most specific Treg marker [41]. Since then, researchers have scrutinized the value of FoxP3 Tregs to determine a potential novel therapeutic target and a prognostic factor in different types of cancer with inconsistent results, especially in malignant melanoma patients [42,43,44,45,46,47].

In this study, we analyzed patients with pT3-stage malignant melanoma treated exclusively in a comprehensive cancer center, and the primary objective was to evaluate whether the overexpression of Tregs FoxP3 correlates with tumor progression and poor overall survival. The secondary objectives were to assess the potential correlations between the expression of Treg Foxp3 and other clinicopathological prognostic elements implicated in malignant melanoma.

## 2. Results

The clinicopathological characteristics of the patients are presented in Table 1. Therefore, 79 patients were included in the analysis, with a median age of 57 years (range 7–80). Ulceration was present in 54 patients, while perineural and angiolymphatic invasion and microsatellitosis were identified in a small proportion. A positive Treg FoxP3 expression was identified in 81% of the cases, with a tumor relapse rate of 36.7%.

Using the univariate analysis, we compared the main clinicopathological features according to the expression of Treg FoxP3, as shown in Table 2.

There was considerable overlap between the two groups regarding the histological subtype of the melanoma, ulceration, angiolymphatic and perineural invasion, microsatellites, and regression, which were findings that were not statistically meaningful (*p* > 0.05). Moreover, the linkage between the expression of regulatory T lymphocytes and tumor infiltrative lymphocytes, which is known to have an essential impact on antitumor immunity and lymph node metastasis rate, was also tested. Therefore, despite the lack of statistical significance (*p* = 0.189), we could observe a higher prevalence of non-brisk peritumoral lymphocytic infiltrate in patients who had a positive expression of Treg FoxP3, in discrepancy to those in the group with a negative expression, in which brisk tumor infiltrative lymphocytes prevailed.

However, we could also find numerous significant associations that might play a paramount role in the evolution of the disease. Consequently, patients who presented a positive Treg FoxP3 expression had a 37% higher risk of developing tumor relapse (*p* = 0.007, 95% CI: 19.56–54.61) and a 12.35 higher rate of death (*p* = 0.004, 95% CI: 1.53–99.6). In addition, we observed a strong correlation between lymph node status and Treg expression, as patients with a negative score developed lymph node metastasis in less than 7% of the cases, in contrast to the other group, with a rate of lymph node metastasis of 50% (*p* = 0.03).

According to the multivariate analysis presented in Table 3, only a positive expression of Treg FoxP3, with an HR of 12.77 (95% CI: 1.67–97.7, *p* = 0.014), and lymph node metastasis, with an HR of 1.81 (95% CI: 1.22–2.68, *p* = 0.003), were associated with a higher risk of death.

**Figure 1:**
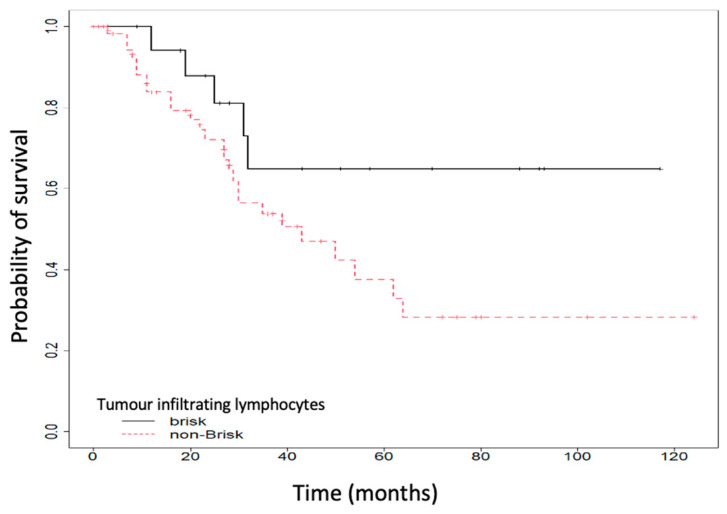
Overall survival (OS) according to tumor-infiltrating lymphocytes (TILs).

We also performed survival analysis using the Kaplan–Meier survival curves. We analyzed the impact of tumor-infiltrating lymphocytes on overall survival. We noticed that patients with a brisk infiltrate had a superior OS compared to those with a non-brisk or absent TILs (64.87% vs. 37.58%, *p* = 0.07, HR 2.36 (96%CI: 0.9–6.16), *p* = 0.08), as shown in Figure 1.

**Figure 2:**
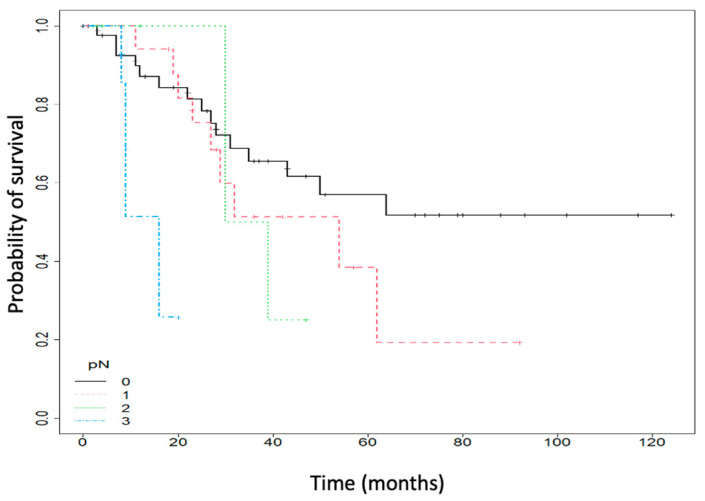
Overall survival (OS), according to lymph node metastasis (pN).

Moreover, patients with no lymph node metastasis had a five-year OS of 56.96% (95% CI: 41.61–77.97), compared to 38.5% (95% CI: 17.82–83.18) for those with one lymph node metastasis and 25% (95% CI: 4.58–100) for those with more than two metastatic lymph nodes. In addition, using a COX regression model, patients with lymph node metastasis had a 9.81 (95% CI: 2.7–35.65) more substantial risk of death compared to patients with no lymph node metastasis (*p* < 0.001). Figure 2 depicts data.

**Figure 3:**
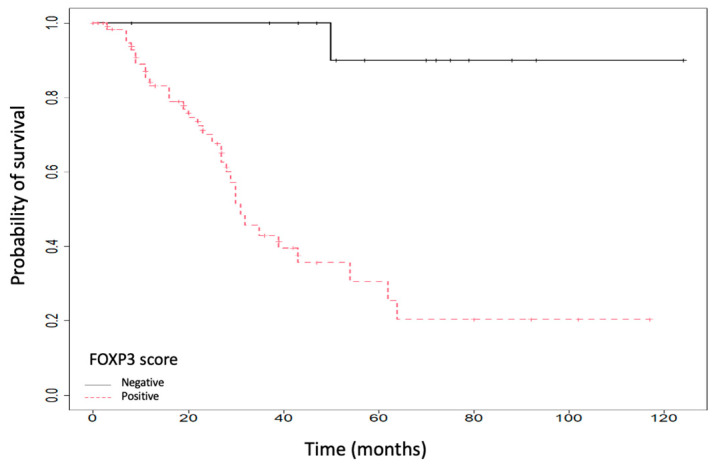
Overall survival (OS) according to TREG FOXP3 (transcription factor Forkhead Box P3) expression.

OS survival was also influenced by the expression of Treg FOXP3, as shown in Figure 3. Therefore, a positive TREG FOXP3 expression was correlated with a lower OS (30.53% (95% CI: 17.84–52.24), *p* < 0.001).

**Figure 4:**
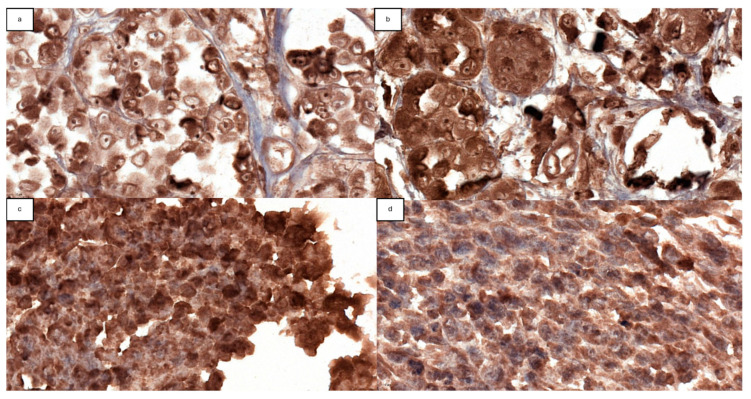
FOXP3 IHC staining; (**a**) minimally positive FOXP3 staining; (**b**) maximally positive FOXP3 staining; (**c**) minimally negative FOXP3 staining; (**d**) maximally negative FOXP3 staining. Scale bar: 10 µm.

Figure 4 shows FOXP3 immunohistochemical staining with respective minimal and maximal visualization.

## 3. Discussion

The current study assesses the expression of Treg FoxP3 in a cohort of stage II and III malignant melanoma patients. It analyzes the association between Treg FoxP3 and the major clinicopathological factors in formulating an accurate prognosis for these patients. The findings validate the existing evidence in the literature, confirming that the immune response plays a fundamental part in developing melanoma and promoting tumor progression. Consequently, an accurate prediction of the prognosis is essential in applying the concept of personalized medicine so that the patient can benefit the most from the current modern therapies. As the plasticity of melanoma cells may lead to an early immune escape and tumor progression, the development of biomarkers is paramount for evaluating the treatment response to improve clinical outcomes.

FoxP3 is a 431 amino acid of the forkhead winged helix family and represents a key regulator for Treg development and function. More specifically, it endows T cells with suppressive functions. It acts as a transcriptional repressor of essential genes involved in T cell activation and functions, including the proliferation and synthesis of proinflammatory cytokines [41,48]. Moreover, the FOXP protein family includes four members (FoxP1, FoxP2, FoxP3, and FoxP4), of which FoxP3 is expressed mainly by CD4+ T lymphocytes, called Tregs; however, a small proportion of T lymphocytes may also express FoxP3 expression, called non-Tregs [49,50]. Also, the following classification of FoxP3 T cells has been proposed: CD45RO+FOXP3high T cells as classical Tregs with suppressive activity, CD45RO+FOXP3low naive Tregs, and CD45RO+FOXP3low non-Tregs in order to better stratify Treg FOXP3 subpopulations, but, due to conflicting results in the literature, it is not routinely used [51,52,53].

T regulatory cells (Tregs) are heterogeneous cells, and different subsets may arise in distinct tissues. Tregs can be classified according to their origin and function as follows: thymic and peripheral Tregs. Thymic Tregs develop naturally by eliciting self-antigens and costimulatory molecules in the thymus and exert an inhibitory effect through intercellular contact [54]. Moreover, Tregs are considered the principal cells responsible for preventing autoimmune diseases. Peripheral Tregs are derived from peripheral memory T cells and induced after exposure to different factors in the tumor microenvironment, including cytokines and tumor antigens [55]. Both types of Tregs express and depend on the FOXP3 transcription factor to effectively suppress immune responses [50]. Thus, a FoxP3 mutation may cause Treg dysfunction, leading to the development of autoimmunity, with diseases such as polyendocrinopathy enteropathy X-linked syndrome, a recessive immune disorder occurring in children [56]. Tregs exert suppressive functions through distinct mechanisms, such as the secretion of proinflammatory cytokines, the expression of co-inhibitory molecules, such as CTLA-4, the modulation of antigen-presenting cells, or the depletion of growth factors from the microenvironment [57].

In tumors, there is a mixture of T cells, including CD4+ and CD8+ cells, in different proportions, depending on the host’s immune status [58,59]. CD8+ T cells exert a cytotoxic effect on tumor cells, whereas CD4+ T cells have immunomodulatory effects by functioning as effector cells and activating cytotoxic T lymphocytes [60,61,62]. In recent years, the expression of FoxP3 Tregs has gained much interest, and several studies were performed to investigate the overexpression of these cells as a prognostic factor in different types of cancers. Therefore, a typical expression of FoxP3 has been identified in normal thymus, lung, breast, and prostate tissue and ovarian epithelium [44]. In contrast, a high expression was described in various types of cancer, including pancreatic adenocarcinoma, malignant melanoma, hepatocellular carcinoma, leukemia, bladder cancer, thyroid cancer, and cervical cancer, with different significance concerning the prognosis and overall survival, having a pro or antitumorigenic role, depending on the tumor type [45,63,64,65,66,67,68].

Considerable investigations scrutinized the potential value of FoxP3 Tregs regarding the prognosis of patients with malignant melanoma. Fuji et al., in a small study in 2011, examined whether there are perturbations of FOXP3 T cells in melanoma, with results revealing that these patients present perturbations of both regulatory and non-regulatory FoxP3 T cells, the degree of perturbations being associated with tumor burden and progression [69]. In addition, Niu et al. assessed the FoxP3 expression in several primary and metastatic malignant melanoma lesions. They heeded that FoxP3 is not expressed by melanocytic nevus, only by melanoma cells, and that the expression induced a suppressive activity on T cells, which may represent a mechanism of tumor resistance to immune destruction [63]. Another study published in 2013 by Gerber et al., including 185 patients, analyzed the influence of FoxP3, CD1a, and langerin expression on the prognosis of primary melanoma patients. According to their results, disease-free survival and overall survival were significantly longer in patients with a low expression of Treg FoxP3. In contrast, a high FoxP3 expression was correlated with a reduced overall survival, independent of tumor thickness. Also, the authors suggest that the suppression of the immune response is crucial during the formation of distant metastasis. Therefore, immunosuppression is a pivotal process in melanoma progression [49]. In addition, Ryan et al. assessed, in a study, an immunoreactivity score (Modified German Immunoreactive Scoring System based on the intensity of staining and the prevalence of positive cells) in patients with head and neck melanomas. They confirmed that the FoxP3/indoleamine 2,3-dioxygenase immunoreactivity score correlates with lymph node positivity and poor outcome [70]. In our study, we proved that patients with a high FoxP3 expression are correlated with a higher rate of lymph node metastasis, a higher rate of recurrence, and a decreased overall survival, validating the literature findings.

On the other hand, in a study that included 146 patients with stage III and IV melanomas, the authors identified FoxP3 expression in only 12% of the cases. They observed that a FoxP3 overexpression suppressed proliferation and increased differentiation and apoptosis, reducing tumorigenesis. Therefore, they concluded that FoxP3 is not likely to be a vital tumor suppressor or promoter in melanoma. Moreover, the authors compared different commercially available antibodies to examine FoxP3 protein expression. They highlighted the significance of this aspect, concluding that the 236A/E7 clone is the most suitable antibody for the immunohistochemical detection of FoxP3 expression [44]. Our study used the clone mentioned above to determine the FoxP3 expression to obtain accurate information and avoid any biases.

In a systematic review and meta-analysis including 76 articles with 17 different types of cancer, Shang et al. observed, according to the pooled analysis, that a high expression of FoxP3 Tregs harmed patient survival, being significantly associated with a decreased overall survival (OR 1.46, *p* < 0.001) in cancers such as cervical, renal, and breast cancers and malignant melanoma. On the other hand, a high FoxP3 Treg infiltration was also associated with improved survival in colorectal, head and neck, and esophageal cancer. Therefore, the authors concluded that the prognostic value of Treg FoxP3 is influenced by the tumor site, mainly correlated with the tumor stage and molecular subtype. Moreover, they emphasized that the differences regarding the prognosis in different cancers might arise from different biological properties of specific tumor types [48]. Moreover, Leslie Connull conducted a study that investigated the density of Treg FoxP3 concerning BRAF and Nras mutations. The authors reported no correlations between Nras mutation and Foxp3 expression; however, a higher density of FoxP3 staining cells was observed among BRAF mutant and wild-type melanomas, correlated with a higher rate of metastasis. In addition, the authors assessed whether the FoxP3 Treg density in the primary tumor might predict the response to BRAF inhibitor therapy but found no statistically significant correlations [42]. A few years later, another study verified the prognostic role of tumoral PDL1 expression and peritumoral FoxP3 lymphocytes in vulvar melanomas and included 75 primary malignant melanomas. They observed that an increased density of peritumoral FoxP3 lymphocytes had a positive impact on survival, independent of tumor thickness, and a PDL1 expression correlated with tumoral and peritumoral CD8+ and FoxP3 lymphocytes; the significance of these findings is that vulvar melanomas, close to the mucosal region, may exert different characteristics from cutaneous melanomas, and patients may have increased survival, with a definite benefit from immunotherapy with anti-PDL1 agents [71].

In another systematic review and meta-analysis, the authors included 72 articles and reviewed the prognostic roles of TIL responses and FoxP3+ TILs in melanoma prognosis. Therefore, the presence of TILs (brisk infiltrate) was correlated with a lower rate of relapse (RFS) in three studies [HR: 0.72 (0.58–0.90)], with a higher rate of DSS [HR: 0.46 (0.30–0.70)] and a higher overall survival in twelve studies [HR: 0.61 (0.52–0.72)]. Regarding the value of FoxP3 TILs, nine studies indicated a better overall survival in patients with high FoxP3 TILs infiltration [HR: 0.57 (0.40–0.82)], but no studies reported data on DFS and DSS. Moreover, the authors stated that the studies included in the metanalysis involved cutaneous melanomas, as none included uveal or mucosal melanomas [43].

Our study assessed the prognostic value of Treg FoxP3 expression in patients with pT3 cutaneous malignant melanomas. We observed that a positive expression of Treg Foxp3 is correlated with a higher rate of lymph node metastasis and a higher rate of recurrence. These findings concur with the available studies in the literature. Moreover, FoxP3 expression negatively influences overall survival, as patients with a positive expression had a poorer outcome and decreased overall survival.

Certain limitations of the current study should be described. First, a retrospective study may contain errors when selecting and including the patients in the database. To avoid this, all the histopathology reports were revised by a different pathologist. Second, our study included a small cohort of patients, with the strict selection of only pT3 tumors, as we consider the melanomas in this stage to have unpredictable behavior and to be difficult to treat, with high rates of tumor progression and rapid relapse. Moreover, to avoid potential biases, we excluded the cases in which immunohistochemical staining with monoclonal antibodies did not appear accurate. Also, we included only cutaneous primary melanomas in the analysis; thus, the prognostic value of Treg FoxP3 expression might be different in uveal or mucosal melanomas. Therefore, even if our study includes a small cohort of patients, we consider it relevant for primary cutaneous melanomas, as it is unicentric, and all the patients were treated after the same protocols, both for surgical approach and adjuvant therapy.

## 4. Materials and Methods

### 4.1. Patient Selection and Data Collection

We analyzed 1842 patients with histologically confirmed malignant melanoma admitted at the Institute of Oncology “Prof. Dr I. Chiricuta” from Cluj-Napoca, Romania, a comprehensive cancer center. We retrospectively conferred the institutional databases over 15 years, from January 2000 to December 2015, and 114 patients were finally included in the analysis. The disease was staged using the latest staging system by the American Joint Committee on Cancer (AJCC, 8th edition, 2018) [72]. The patients were considered eligible for this study based on the following inclusion criteria: (i) patients with histologically confirmed malignant melanoma; (ii) patients who have undergone surgery exclusively at our cancer center; (iii) patients with stage II and III malignant melanoma (pathological T3 staging, with or without lymph node involvement; Breslow thickness 2.01–4.00 mm); (iv) patients who were not previously diagnosed with another malignancy; and (v) patients with adequate follow-up. A total of 35 patients, who were initially included in the analysis, were excluded due to inappropriate immunohistochemistry staining, which might lead to a bias when interpreting the results. The patient treatment and follow-up were conducted according to the institutional melanoma guidelines, which aligned with EU recommendations (ESMO) [73]. Patients treated in other cancer centers or those not following the standard protocols were excluded from this study. Lymph node status was evaluated using surgery, either through direct lymph node dissection (LND) or sentinel lymph node biopsy (SLNB), and completed with lymph node dissection if positive within two months maximum from diagnosis. All study participants provided informed consent on admission, and all clinical information for each patient was coded; finally, the institutional ethical committee approved this study.

### 4.2. Immunohistochemistry Analysis

The pathology slides of the 114 patients were re-evaluated by a proficient pathologist, selecting the area of interest, which was later identified on the corresponding paraffin block. The tumor fragments were included and resettled in a smaller number of paraffin blocks, called tissue microarray (TMA), an economical method that allowed the analysis of multiple biological samples. Thus, approximately 2 mm tissue samples were taken, from which 3 TMA blocks were made. Subsequently, they were placed at 56–58 °C for 20 min, and then the tissue was sectioned, and 4 μm fragments were obtained, placed on slides, and incubated overnight at 37 °C. The Leica Bond III device, Leica Biosystems, was used for immunohistochemical staining. Sections were double-stained with two antibodies as follows: FOXP3 (anti-FoxP3 antibody mouse IHC P [236A/E7]—Abcam—ab20034) as the primary antibody and NovoCastra NovoLink Polymer as the secondary staining antibody. Afterward, each staining was checked to be within optimal parameters, excluding 35 patients whose stainings were not optimal. To avoid possible biases, the slides were scanned with the help of a microscope to be analyzed. The analysis of Treg FoxP3 expression was carried out with the help of the Image J—IHC Profiler program (single version, 09-05-2014), which performs a quantitative evaluation, generating an automatic immunohistochemistry score based on an algorithm with a prediction of 88.6% [74] as follows: intensely positive, positive, weakly positive, and negative. Due to the variability of the results, we considered a positive expression in patients who had an intensely positive and positive score, respectively, and a negative expression in those with a weak positive and negative score, obtaining a combined score in the form of the following 2 variables: positive expression and negative expression.

### 4.3. Statistical Analysis

Qualitative data were described numerically by number and by percentage in column-type graphs. Quantitative data were described by mean and standard deviation or by median, quartiles 1 and 3. The association between qualitative variables was evaluated using contingency tables, with absolute frequencies and percentages on lines, and mosaic/bar graphs, and the χ^2^ test or the exact Fisher test tested the association’s existence. When the relationship between dichotomous qualitative variables was evaluated, the following indicators were used that quantify the importance of the association: odds ratio and attributable risk, with 95% associated confidence intervals. Logistic regressions analyzed the evaluation of the multivariate association. For multivariate models, the presence of multicollinearity was evaluated. The fit of the models was evaluated with the Hosmer–Lemeshow test, and the model’s predictive capacity was evaluated using the overall percentage of correct classification and the area under the receiver operating characteristic. The results are expressed with a 95% confidence interval. The Mann–Whitney U test was used to assess whether there were differences between two independent groups of quantitative data. The difference between the medians was presented to quantify the differences between the groups, with a 95% associated confidence interval.

Survival data were described by presenting the number of events, censored data, and the percentage of survival at five years graphically using Kaplan–Meier survival curves. Comparisons between groups targeting survival data were made using the log-rank test. The hazard rate was calculated for various explanatory variables to assess their association with survival using Cox regression, providing the hazard ratio with the associated 95% confidence interval. The presumption of proportional hazard was verified graphically with Schoenfeld residuals and a statistical test. For all tests, the value of 0.05 was used as a significance threshold, and the bilateral *p*-value was taken into account in the tests that provided it. The statistical environment for statistical calculations and graphs R version 4.0.2 was used for statistical processing.

## 5. Conclusions

A total of 81% of the patients presented a positive Treg FoxP3 expression, which is correlated with a higher risk of lymph node metastasis, tumor relapse, and death. Moreover, positive expression was statistically associated with a shorter OS. The tumor relapse rate was estimated at 36.7%. A positive expression of Treg FoxP3 and lymph node metastasis were associated with a higher risk of death based on multivariate analysis. In conclusion, the expression of Treg FoxP3 significantly influences the prognosis of malignant melanoma and may be used as a biomarker for primary cutaneous melanomas. Moreover, FOXP3 immunohistochemical staining is easy to perform. It may be employed to identify and better stratify the management of patients with a high risk of developing lymph node metastasis, tumor progression, and disease recurrence.

## Figures and Tables

**Table 1:** Clinicopathological characteristics of the patients.

Characteristic	Number (%) (n = 79)
Clark scale	2: 3/79 (3.8) 3: 25/79 (31.65) 4: 44/79 (55.7) 5: 7/79 (8.86)
Ulceration (Yes vs. No)	54/78 (69.23)
Perineural invasion (Yes vs. No)	4/78 (5.13)
Angiolymphatic invasion (Yes vs. No)	8/78 (10.26)
Regression (Yes vs. No)	24/78 (30.77)
TILs ^a^ (brisk vs. non-Brisk)	20/79 (25.32)
Microsatellitosis (Yes vs. No)	13/78 (16.67)
pT ^b^ (3a vs. 3b)	27/79 (34.18)
pN ^c^	0: 46/79 (58.23) 1: 18/79 (22.78) 2: 8/79 (10.13) 3: 7/79 (8.86)
FOXP3 ^d^ expression (Negativ vs. Pozitiv)	15/79 (18.99)
Tumor relapse (Yes vs. No)	29/79 (36.71)
Death (Yes vs. No)	31/79 (39.24)

^a^ TILs = tumor-infiltrating lymphocytes. ^b^ pT = pathologic staging of tumor. ^c^ pN = pathologic staging of lymph nodes. ^d^ FOXP3 = transcription factor Forkhead Box P3.

**Table 2:** Univariate analysis according to Treg FoxP3 expression.

FOXP3 ^a^ Expression	Negative (n = 15)	Positive (n = 64)	*p*-Value
Histological type, number (%)	Acral: 0 (0) Nodular melanoma: 1 (6.67) Superficial spreading melanoma: 14 (93.33)	Acral: 1 (1.56) Nodular melanoma: 14 (21.87) Superficial spreading melanoma: 49 (76.56)	0.707
Ulceration (Yes), number (%)	12 (80)	42 (66.67)	0.37
Perineural invasion (Yes), number (%)	1 (6.67)	3 (4.76)	1
Angiolymphatic invasion (Yes), number (%)	0 (0)	8 (12.7)	0.342
Regression (Yes), number (%)	5 (33.33)	19 (30.16)	1
TILs ^b^ (brisk), number (%)	6 (40)	14 (21.88)	0.189
Microsatellitosis (Yes), number	2 (13.33)	11 (17.46)	1
Melanoma site, number (%)	Head and neck: 3 (20) Arms and legs: 8 (53.33) Thorax and abdomen: 4 (26.67)	Head and neck: 14 (21.88) Arms and legs: 19 (29.69) Thorax and abdomen: 31 (48.44)	0.191
Tumor relapse (Yes), number (%)	1 (6.67)	28 (43.75)	0.007
Death (Yes), number (%)	1 (6.67)	30 (46.88)	0.004
pN ^c^, nr (%)	0: 14 (93.33) 1: 1 (6.67) 2: 0 (0) 3: 0 (0)	0: 32 (50) 1: 17 (26.56) 2: 8 (12.5) 3: 7 (10.94)	0.03

^a^ FOXP3 = transcription factor Forkhead Box P3. ^b^ TILs = tumor-infiltrating lymphocytes. ^c^ pN = pathologic staging of lymph nodes.

**Table 3:** Multivariate analysis.

	HR ^a^ Unadjusted	(95% CI)	*p*-Value	HR Adjusted	(95% CI)	*p*-Value
Breslow index (mm)	0.96	(0.58–1.59)	0.87	0.96	(0.57–1.62)	0.887
Ulcerative (Yes vs. No)	0.52	(0.26–1.06)	0.073	0.72	(0.34–1.55)	0.407
TILS ^b^ (brisk vs. non-Brisk)	0.42	(0.16–1.11)	0.08	0.72	(0.26–2.04)	0.542
pN ^c^	1.81	(1.22–2.68)	0.003	1.38	(0.92–2.07)	0.119
FOXP3 ^d^ score (Positive vs. Negative)	17.34	(2.34–128.6)	0.005	12.77	(1.67–97.77)	0.014

^a^ HR = hazard ratio. ^b^ TILs = tumor-infiltrating lymphocytes. ^c^ pN = pathologic staging of lymph nodes. ^d^ FOXP3 = transcription factor Forkhead Box P3.

## Data Availability

Data are available upon reasonable request from the corresponding author.
